# Expert opinion on a consensus-based checklist for the critical appraisal of cost-of-illness (COI) studies: qualitative interviews

**DOI:** 10.1017/S0266462323000181

**Published:** 2023-06-09

**Authors:** Lena Schnitzler, Aggie T.G. Paulus, Silvia M.A.A. Evers, Tracy E. Roberts, Louise J. Jackson

**Affiliations:** 1 Department of Health Services Research, Care and Public Health Research Institute (CAPHRI), Faculty of Health, Medicine and Life Sciences (FHML), Maastricht University, Maastricht, The Netherlands; 2 Health Economics Unit, Institute of Applied Health Research, College of Medical and Dental Sciences, University of Birmingham, Birmingham, UK; 3 School of Health Professions Education (SHE), Faculty of Health, Medicine and Life Sciences (FHML), Maastricht University, Maastricht, The Netherlands; 4 Trimbos Institute, Netherlands Institute of Mental Health and Addiction, Utrecht, The Netherlands

**Keywords:** Cost-of-illness, checklist, critical appraisal tool, interviews, qualitative analysis

## Abstract

**Objectives:**

This study explored experts’ views on the development of a proposed checklist for cost-of-illness (COI) studies. It also investigated experts’ perspectives on the use of COI studies and quality/critical appraisal tools used for COI studies as well as their experiences with the use of these tools.

**Methods:**

Semi-structured, open-ended interviews were conducted with health economists and other experts working with COI studies and with experience of developing health economic guidelines or checklists. Participants were selected purposively using network and snowball sampling. A framework approach was applied for the thematic data analysis. Findings were reported narratively.

**Results:**

Twenty-one experts from eleven different countries were interviewed. COI studies were found to be relevant to estimate the overall burden of a disease, to draw attention to disease areas, to understand different cost components, to explain cost variability, to inform decision making, and to provide input for full economic evaluations. Experts reported a lack of a standardized critical appraisal tool for COI studies. Their experience related predominantly to guidelines and checklists designed for full economic evaluations to review and assess COI studies. The following themes emerged when discussing the checklist: (i) the need for a critical appraisal tool, (ii) format and practicality, (iii) assessing the questions, (iv) addressing subjectivity, and (v) guidance requirements.

**Conclusions:**

The interviews provided relevant input for the development of a checklist for COI studies that could be used as a minimum standard and for international application. The interviews confirmed the important need for a checklist for the critical appraisal of COI studies.

## Background

Cost-of-illness (COI) studies aim to assess the economic burden of an illness (or disease, health condition, risk factor) on society (1). This generally involves the identification, measurement, and valuation of healthcare costs and/or non-healthcare costs across different sectors of society (e.g., intersectoral costs), depending on the study perspective (e.g., healthcare, societal). For example, a societal perspective is expected to capture all relevant costs associated with an illness both within and beyond the health sector (e.g., productivity losses and informal care) (2).

An accurate estimation of COI is essential to optimally inform different stakeholders. In the context of health policy/decision making, this can be essential to prioritize certain health interventions or policies and to allocate resources accordingly and under budget constraints (2;3). In health economics research, this can be relevant to provide adequate information on valuable cost estimates, for example, to inform the conduct of full economic evaluations (4–8). This requires COI studies to be methodologically sound and to be as comprehensive and transparent as possible.

There is, however, an extensive methodological heterogeneity among COI studies due to poor consensus on methodological approaches and a lack of a standard tool to review and assess COI studies in terms of their comprehensiveness, transparency and consistency (6;9–13). Different methodological approaches can be found, for instance, in relation to the objective(s), study perspective(s), and costs captured. Heterogeneity can also be a result of the different data sources available for COI studies. Such heterogeneity can hinder comparison and transferability of study results (10;14). This highlights the important need for a standard tool to critically appraise COI studies that could be used as a minimum standard.

The controversy around COI studies and its importance for research and policy/decision making is briefly described below before presenting the objectives of the study and methodological approaches.

### Controversy around COI studies

Although COI studies have an important role to play in health economics and are a useful economic tool for policy/decision making, these studies have been the subject of extensive debate. One argument against COI studies that is often articulated is that they simply identify an area of high expenditure and could lead to those illnesses being focused on that are the most costly (15), rather than those that are judged as the highest priority.

The controversy on the usefulness of COI studies largely concerns its methodology. They are criticized for only considering the costs of resources and not the utility loss associated with an illness. It is argued that COI studies do not capture information on the benefits (outcomes) associated with interventions and that the lack of understanding and comparison of the costs and benefits makes it impossible to determine whether resources are being spent efficiently (16). This controversy has resulted in COI studies being overlooked and their role as an important economic tool being questioned (15).

However, a number of alternative arguments have been put forward in relation to the importance of COI studies, in particular in relation to the true total cost to society (15). First, COI studies reveal relevant information on how much society is spending on an illness. COI estimates can be used as a foundation for projecting disease expenses or a framework to address a certain health problem (6;15). Second, COI studies are used to provide policy/decision makers with information regarding the different cost components and cost categories (or sectors) associated with an illness. It is argued that identifying the main cost components is essential in order for policy/decision makers to define cost containment strategies, in particular for main cost drivers (15). The consideration of both health and non-health (e.g., intersectoral) costs (or resources) is crucial to reflect the most comprehensive total costs to society. Unless both health and non-health costs are captured total cost estimates can be insufficient, and this can lead to suboptimal decision making (17). A recent systematic review of COI studies found that the intersectoral costs associated with sexually transmitted infections and HIV can largely contribute to the total economic cost burden of those diseases (18). Further, findings arising from COI studies can be used as a justification of the necessity associated with health interventions or policy (2;5). COI estimates are also needed to inform decision analytic modeling on the cost-effectiveness of interventions (5;6;19).

This study aimed to explore experts’ views on the development of a proposed checklist for COI studies developed by the author team https://doi.org/10.1017/S0266462323000193, using qualitative interviews. It also investigated experts’ perspectives on both the use and relevance of COI studies and of quality/ critical appraisal tools used for COI studies as well as their experiences with the use of existing quality/ critical appraisal tools. In this study, we use the term “experts” to refer to individuals that are knowledgeable in a particular area, in this case in health economics/COI studies, and are/were actively involved in doing research around COI studies.

This paper is complementary to the study that published the consensus-based checklist for COI studies and provides a broader context and in-depth analysis of the interviews https://doi.org/10.1017/S0266462323000193.

## Methods

Semi-structured, open-ended interviews were conducted with health economists and other experts working with COI studies and with experience developing health economic guidelines or checklists, to seek their opinion on the relevance and use of COI studies, existing quality/ critical appraisal tools for COI studies, and to contribute to the development of a checklist for COI studies.

### Sampling and recruitment of participants

Interview participants were selected purposively based on their expertise in health economics, their knowledge on COI studies and their experience in developing health economic guidelines or checklists. Using network and snowball sampling, experts were approached via E-mail and invited to participate in a one-to-one online interview with the lead researcher (LS). An information leaflet was shared with all potential participants to provide more information on the background and purpose of the study and the dissemination of study findings. Before their interviews, participants received the preliminary checklist for COI studies developed by the author team and a consent form (Supplementary Table S1).

### Data collection and analysis

A semi-structured approach was chosen for the conduct of the interviews as it allows for a systematic coverage of key topics alongside flexibility and spontaneity (20). Online interviews were chosen due to the circumstances relating to the coronavirus disease-2019 (COVID-19) pandemic. A topic guide was developed to guide the interviews, containing a set of open-ended questions (Supplementary Table S2). Some of the questions involved asking whether participants had used any form of critical appraisal tool or quality assessment(s) for COI studies to date, and if so, why they chose to apply certain tools, and what these entailed. Participants were also invited (i) to share their feedback on the preliminary checklist, (ii) to comment on the relevance of individual questions included in the checklist, (iii) to suggest additional questions, and (iv) to state whether the checklist was comprehensive. Interviews were audio-recorded, with the participant’s consent, and anonymized.

A Framework approach was applied for the thematic analysis, allowing for systematic analysis (21). The interview recordings were transcribed in full and entered into NVivo 12 (a software for qualitative data analysis) by the lead researcher (LS) (step I). The same researcher (LS) and a second author (LJ) both familiarized themselves with a number of the recordings and transcripts (step II) and independently coded several transcripts, identifying emerging themes and sub-themes (step III). The authors compared their themes and sub-themes, discussed discrepancies, and discussed those themes with all co-authors (step IV). This resulted in a coding framework that all study authors agreed upon. The lead researcher (LS) then coded the remaining transcripts, applying the established coding framework (step V). A matrix was developed, charting all themes and sub-themes found in all transcripts (step VI). This was again shared with all co-authors for discussion (step VII). The findings are reported narratively.

### Consent

All interview participants signed and returned their written consent before the start of their interview.

### Ethical approval

Ethical approval to conduct this study was obtained from the University of Birmingham (ERN_20–1240).

## Results

### Study characteristics

Twenty-one experts (eleven male, ten female) with experience of undertaking or working with COI studies from eleven different countries participated in the interviews between October 2020 and April 2021 (Table 1). This involved experts from Europe (*n =* 13), Australia (*n =* 1), Canada (*n =* 1), the Middle East (*n =* 1), and the United States (*n =* 5). Experts had expertise in health economics (*n =* 17), economics, (*n =* 1), health policy (*n =* 2) and psychology (*n =* 1). Most of them were affiliated with academia or research institutes (*n =* 17); others with international policy organizations (*n =* 1), governmental organizations (*n =* 1), and consulting firms (*n =* 2). Three of the experts had experience in developing checklists for health economic studies. The interviews ranged between 45–75 minutes.

**Table 1.** Interview sample

Participants		21
>10 years of experience (approximately)		11
<10 years of experience (approximately)		10
*Country*		
Australia		1
Canada		1
Denmark		1
France		1
Germany		2
Ireland		1
Italy		1
Lebanon		1
Netherlands		1
United Kingdom		5
United States		5
*Expertise*	*Affiliation*	*ID*
Health economics	University/Research Institute	I.1–I.9; I.11–I.15
	International Policy Organization	I.16
	Consultancy	I.17; I.18
Economics	Governmental Organization	I.19
Health policy	University/Research Institute	I.10; I.20
Psychology	University/Research Institute	I.21

### Interview findings

COI studies were generally found to be relevant to estimate the overall burden of a disease, to draw attention to disease areas and their impacts, to understand the different cost components and the total costs, to explain cost variability, to inform decision making, and to provide input for cost-effectiveness analyses.

One expert explained that “we as health economists usually get the request to do cost-of-illness studies to help inform decision-making around new vaccines and whether introducing a vaccine against a disease, or even earlier in the development chain, whether even inventing in R&D in a new vaccine is relevant. […] This is where that cost-of-illness information comes in, as a first step. It’s also used as an input for cost-effectiveness analysis eventually” (Economist, I.19).

Some differing opinions were identified, with one participant raising the concern that COI studies “are often used as an advocacy tool, saying that a specialty is important as it costs a lot of money” (Psychologist, I.21). Another claimed, “Cost-of-illness tends to be used as an attempt to draw attention to a disease in the hope that [it] will encourage a greater amount of funding” (Health Economist, I.15).

Further, experts reported a lack of a standardized critical appraisal tool for COI studies. Their experience with existing quality/ critical appraisal tools primarily related to guidelines and checklists designed for full economic evaluations such as the Drummond Methods for Economic Evaluation in Healthcare and the CHEERS guidelines. A small number of experts were familiar with the Guide for Critical Evaluation by Larg and Moss (28). When assessing COI studies, experts stated they often referred to previous studies or reviews of COI studies to adopt their methodology or their set of questions for quality assessment or critical appraisal. The main purpose for assessing COI studies was as part of a (systematic) review.

The following themes emerged from the interviews when discussing the preliminary checklist: (i) the need for a critical appraisal tool, (ii) format and practicality, (iii) assessing the questions (how to answer them), (iv) addressing subjectivity, and (v) guidance requirements.

#### The need for a critical appraisal tool

The interviews validated that at the time there was no consensus on either a standard guideline for the methodology of COI studies or a standard critical appraisal tool to review and assess COI studies. Experts considered the proposed preliminary checklist to be important to address this gap. Those who previously conducted COI studies explained they relied on their own knowledge and experience or what other researchers have done before in terms of methodology. Applying different methodologies can lead to heterogeneity across studies and difficulties in comparing study findings.

There is no consensus, there is no standard way to think about methodology. People could have different opinions and it’s really hard to say one [study] is better than the other. (Health Economist, I.5)

To begin with, I think it’s brilliant that you have this idea of creating a checklist because there aren’t any. (Psychologist, I.21)

There is not a normative guideline that we use in developing some of the cost-of-illness studies. We are more building on what has been done internally. For example, there has been an influenza economic burden tool developed by WHO and then we used that tool to then do an influenza burden of disease study in other countries. (Economist, I.19)

It was highlighted that guidelines for economic evaluations are often used to conduct COI studies or assess its quality, but that this is methodologically suboptimal.

At the end of the day, we work alongside the guidelines for economic evaluation. However, they don’t really fit. We mostly rely on our own experience, and we look at literature, what other researchers are doing. (Health Economist, I.14)

The important need for a checklist for COI studies specifically was expressed repeatedly.

There is an importance of having a strong checklist and a good guidance in order to assess whether we are working in a methodologically sound way, ‘yes or no’. […] So we need such checklist or guidance in order to know if what we are doing is optimal. (Health Economist, I.4)

#### Format and manageability

The interviews elicited views on the overall format and manageability of the checklist. It was suggested to keep it concise, and experts felt that a balance was needed between being comprehensive and ensuring the checklist was practical for use.

First, I certainly understand the thought behind developing this checklist. […] It is not appreciated to have to use a long checklist when having to evaluate existing studies. […] It is not practical. I think it makes sense to cut down on criteria as you did with the [CHEC-list]. (Health Economist, I.12)

The checklist is high-level. I think that the way that you drafted it is OK. Otherwise, you need to go too much into detail. […] and this can become too cumbersome. (Health Economist, I.9)

I prefer a shorter checklist and if there are a lot of ‘no’s’ you kind of take a closer look and see what exactly is going on here, also in terms of limitations. (Health Economist, I.2)

Only a minority of the experts suggested to add more directed and technically detailed questions but argued that this was primarily helpful to guide the conducting of COI studies.

But maybe it’s a bit short because if you conduct a cost-of-illness study it comes down to very specific questions. (Health Economist, I.14)

I mean it’s a quality assessment tool, it’s not a guideline. I think guidelines are more detailed and explain why you have to choose certain approaches. I don’t think that’s necessary for a checklist. […] I don’t think you have to be too detailed in a checklist, details are for guidelines. (Health Economist, I.14)

Dividing the questions listed in the checklist by domain (study characteristics, methods and data analysis, results and reporting) was welcomed by the experts.

I think it’s nice and simple. The main, top level categories, study characteristics, methods, results and reporting […]. This is following pretty much the economic evaluation kind of checklists. I think the questions are all relevant, I can’t see any here which are not needed. (Health Economist, I.16)

It’s clear first of all, and it’s good that you divided it based on study characteristics, methodology and cost analysis, this is very helpful. (Health Economist, I.4)

#### Assessing the questions (how to answer them)

A discussion evolved in many interviews around how to answer the questions in the checklist. There was consideration of whether the response should be scored or simply an assessment of whether an item was present or absent.

When I saw your checklist my first reaction was ‘how are you going to score each of the questions?’ […] Nobody knows what the most ideal way is of doing the scoring. (Health Economist, I.5)

Most experts suggested to avoid applying a numerical score when assessing the questions (e.g., yes = 1, no = 0). The main argument was that not all questions are equally important and have the same weight.

But the main issue is how to score the items. And second, are the items given the same weight. Are there any relatively more important items. […] Or are there more important items that truly affect the results of the study. (Health Economist, I.4)

As part of one of our reviews we were thinking on whether we want to add a score and add up the scores, but we didn’t because working with scores is not appropriate. Because we were unable to decide which questions should be given more weight compared to others. (Health Economist, I.14)

I’m always a bit more cautious about giving a score in a checklist because that implies something about how important each criterion is compared to another. A scoring process is another piece of work, so I guess I’m against scoring. (Health Economist, I.6)

We saw in the pre-test of our checklist that people wanted a quantification, so a total score. […] But this is not in the nature of cost-of-illness studies. Cost-of-illness studies might have scored well on some criteria but should the one or two criteria that they didn’t score well on really ruin the quality of the study. In that case a score can be treacherous. (Health Economist, I.12)

There was some controversy around whether the answers to questions should be limited to just ‘yes-or-no’ responses, with some experts arguing in favor of this.

My preference has been yes-or-no and have an accumulative score. But I don’t know if all of these elements are equally weighted. (Economist, I.19)

A checklist with a yes-or-no process would actually be quite useful because I could get quite a lot from that checklist quite quickly. (Health Economist, I.6)

Whereas many others felt that a more nuanced response would be needed. As they felt that some aspects might be very clear, but others might be less well covered.

For example, ‘The research question was posed in an answerable form’. It might be clear to some, it might not entirely be clear to others, so a yes or no could be a bit too strict. (Health Economist, I.9)

Question 2 is a yes-or-no question and what do I do if my answer is somewhere between 1 and 2. (Health Economist, I.12)

It was also argued that yes-or-no answers would be unfair for those studies that did not have all the necessary resources or data available for the COI study.

Maybe there should be a place somewhere in the middle because for us as low-middle income-country sometimes you don’t find the source of the costs or you are expected to value them in a way that is not optimal but at least you did something. So yes-or-no answers are a bit harsh. (Health Economist, I.4)

Instead, intermediate answer categories were suggested for use to avoid misjudgment when an answer is not clear.

Thinking about whether it makes sense to include intermediate answer categories. I always think it makes sense because there are often questions where the answer is not clear. Similar to the COCHRANE risk of bias tool with low, medium and high risk. […] I find it difficult if the in-between answers are not provided or not an option. If I have to decide on whether it is a yes or no, this may risk a misjudgment. (Health Economist, I.12)

It doesn’t have to be numerical scoring. For example, the GRADE one where you have low, medium and high risk and at the end of your review you can give the reader an understanding of what is the quality of the literature. (Health Economist, I.7)

Perhaps I would prefer it kind of a gradient scale instead of saying yes-or-no. […] A scale could be more flexible and could also provide the reviewers with more opportunities to express their opinion about the study. (Health Economist, I.9)

The idea of adding a data extraction column to the checklist was expressed. This would allow the users of the checklist to add supportive information to their answers, which in turn could enhance accountability.

I do think it makes sense to have a separate column to fill in your answers much like a data extraction. (Health Economist, I.12)

In the PRISMA checklist it is like that, adding a page number or sometimes you have to add an excerpt depending on the journal. But I do think that this really forces accountability. (Economist, I.19)

That’s a problem with the CHEERS checklist, it’s incredibly long and it gets very tedious. […] I quite like the idea of having a data extraction column and putting the information in there rather than referring to that sentence on a page number. (Health Economist, I.1)

#### Addressing subjectivity

Questions should be as clear as possible, as suggested by experts, to avoid or reduce subjectivity. There was particular concern about the interpretation of the word ‘appropriate’ which could be interpreted in different ways.

People have different ideas of what is appropriate. People would argue vehemently for a friction approach to measure productivity losses. Other people would argue vehemently the human capital approach is appropriate. And they’re going to give completely different estimates. (Health Economist, I.17)

Appropriateness; you talk about whether it’s relevant or not. That requires interpretation and so the difficulty is, it’s not necessarily repeatable. (Health Economist, I.2)

Obviously the tricky part is how do you gauge whether someone did something appropriately or not versus they are just limited to what they are doing. Especially if you start to think about a societal perspective, it gets tricky. At some level you’re going to have to cut it even though you are adopting a societal perspective. […] Obviously, people have their own incentives in terms of thinking about whether these are appropriate versus not appropriate. (Health Policy, I.20)

Suggestions were given by experts to rephrase some of the questions to avoid such subjectivity, such as:

Sometimes it’s not really clear what you mean by appropriate. I would probably use something more like ‘Was the study design described or motivated?’. (Health Economist, I.3)

It’s very subjective to use the term ‘appropriate’, I would just ask ‘Is the study design stated?’. (Health Economist, I.14)

#### Guidance requirements

The inclusion of guidance statements to explain each of the questions was recommended. This was suggested as important to give further detail and provide examples of best practice. Guidance statements were also seen as helpful in reducing subjectivity in answers.

You might need an accompanying piece to your checklist and explain the questions, like a table that explicates what each of the questions are really getting at and with an example. Because unless you give people a tool that says here is an example of appropriate […], you just can’t answer it. (Economist, I.19)

If you kept the questions like this you can have another paper of what you actually mean by the questions. (Health Economist, I.7)

When you have an article that goes through these different domains and criteria and then at the end almost have an appendix or a table where you’d have the checklist. (Health Economist, I.16)

## Discussion

### Principal findings

This study is the first to explore experts’ views to inform the development of a checklist for COI studies in English and to simultaneously investigate experts’ perspectives on the use and relevance of COI studies and critical appraisal tools for COI studies (please refer to the companion paper for more detailed information on how the checklist was developed).

The research findings highlight that at the time of the interviews there was no standard, consensus-based checklist for the critical appraisal of COI studies available in English. This risks inconsistency in the methodology across COI studies and increases heterogeneity. Consequently, optimal comparability across study findings and transferability of results are challenging, if not impossible (9;10;12;13;22–26). This study gathered data on the relevance and need for such checklist as well as what the checklist would need to entail to be considered comprehensive, practical, and a minimum standard for use. It presents findings about the format, ways of assessment, wording, and guidance requirements such a checklist should best fulfill.

Overall experts appreciated that the checklist was short but comprehensive. A longer and more technically detailed checklist was seen as burdensome, in particular, when having to apply it to a larger number of studies as part of a review. Opinions differed slightly regarding how to best to answer the questions in the checklist. There was general agreement among the participants around the need to provide guidance statements explaining each question to help avoid potential misunderstanding and to reduce subjectivity.

### Comparison with other studies

Methods adopted by other existing studies to develop checklists/guidance were taken into consideration for the development of the present checklist (27–29). Like this study, previous studies made use of stakeholder interviews or Delphi panels to develop guidelines or checklists for full economic evaluations including the CHEC-list (27;30;31). To the best of our knowledge, only one other study exists that developed a checklist specifically for COI studies and incorporated stakeholder interviews in the development process; the checklist by Müller et al. (29). However, their checklist was established for the German context and is officially only published in German. The Guide to Critical Evaluation by Larg & Moss (28) was also designed for COI studies, but it is not clear whether they had considered expert opinion as part of their development process (32).

### Strengths and weaknesses of the study

This paper provides a thorough overview of experts’ perspectives elicited from qualitative interviews. Participants had direct experience of working with COI studies, undertaking COI studies, developing health economic guidelines and checklists, and applying such; which is a key strength of this study. The involvement of consultations with 21 experts from eleven different countries with professional experience in health economics, economics, health policy, and/or psychology, and with a range of different affiliations, adds to the strengths of this study. Another strength is the use semi-structured, open-ended interviews, which allowed for a structured approach to guide the interviews as well as the opportunity for new themes to emerge and be explored. The author team acknowledges that there could inevitably be some limitations associated with the study. There might be additional considerations regarding the checklist that were not captured, and it is suggested that future research is undertaken to explore such factors. In particular, as the interviews were conducted with participants based in OECD countries, it would be important to capture the views and experiences of those based in other settings. Another limitation could be that experts might not have felt comfortable stating that they did not feel there was a need to develop and implement a checklist for COI studies given the focus of the PhD research. However, interviewees were encouraged to provide their honest professional opinion both before and throughout the interviews.

## Conclusion

The interviews provided relevant input for the development of a consensus-based checklist for COI studies that could be used as a minimum standard and for international application. The interviews also confirmed the important need for a checklist for the critical appraisal of COI studies.

## Supplementary material

For supplementary material accompanying this paper visit https://doi.org/10.1017/S0266462323000181. click here to view supplementary material
