# Supplementary material for: Expert opinion on a consensus-based checklist for the critical appraisal of cost-of-illness (COI) studies: qualitative interviews
Source: Int J Technol Assess Health Care. 2023 Jun 9;39(1):e33. doi: 10.1017/S0266462323000181 (PMC11574535; doi:10.1017/S0266462323000181)
Supplement: Supplementary file 1 [file S0266462323000181sup.zip › S0266462323000181sup001.docx]

Supplementary table 1

Preliminary checklist for COI studies

| Study characteristics |
| --- |
| 1. Is the study population clearly described? |
| 1. Is a well-defined research question posed in answerable form? |
| 1. Is the economic study design appropriate to the stated objective? |
| 1. Is the chosen time horizon appropriate in order to include relevant costs? |
| 1. Is the actual perspective chosen appropriate? |
| Methodology and cost analysis |
| 1. Are all important and relevant costs identified? |
| 1. Are all costs measured appropriately? |
| 1. Are costs valued appropriately? |
| 1. Are all future costs discounted appropriately? |
| 1. Are all important variables, whose values are uncertain, appropriately subjected to sensitivity analysis? |
| Results and reporting |
| 1. Do the conclusions follow from the data reported? |
| 1. Does the study discuss the generalizability of the results to other settings and patient/client groups? |
| 1. Does the article indicate that there is no potential conflict of interest of study researcher(s) and funder(s)? |
| 1. Are ethical and distributional issues discussed appropriately? |
| 1. Does the study discuss any limitations (i.e., cost components, data assumptions, methods)? |
| *Question 1-14: based on the CHEC-list (2005); Question 15: adopted from Larg & Moss (2011)* |
