# Supplementary material for: Expert opinion on a consensus-based checklist for the critical appraisal of cost-of-illness (COI) studies: qualitative interviews
Source: Int J Technol Assess Health Care. 2023 Jun 9;39(1):e33. doi: 10.1017/S0266462323000181 (PMC11574535; doi:10.1017/S0266462323000181)
Supplement: Supplementary file 1 [file S0266462323000181sup.zip › S0266462323000181sup002.docx]

Supplementary table 2

Interview topic guide

*Introduction*

- Can you shortly describe your current profession?
- How long have you been working in health economics?

*COI studies and quality appraisal tools*

- Do you currently work with COI studies?
- In your opinion, why are COI studies important (or not important)?
- In your opinion, what can COI studies be used for? What is the objective?
- What is your professional opinion on the use of COI studies in health economics and in health policy?
- Are you familiar with a guide for COI studies?
- Are you familiar with a quality appraisal tool for COI studies?
- In your opinion, what is the main purpose of conducting a quality appraisal of COI studies?
- Do you currently work with a quality appraisal tool for COI studies (or have you recently?)
- If so, which tools (or checklists) have you worked with so far to assess the quality of COI studies? Why did you choose this checklist or tool? How did you hear about this tool or checklist?
- If not, where would you search for a quality appraisal tool for COI studies, if you sought one?
- Are there any other tools or checklists you know of that could be used for the quality assessment of COI studies?
- In your opinion, what would an ideal checklist entail when assessing the quality of COI studies?
- How would you design it?
- Where would you start looking for and developing a new checklist for the quality appraisal of COI studies?

*Feedback on the preliminary checklist*

- Having had the opportunity to look at the preliminary checklist - what are your thoughts on the checklist in general?
- What are your thoughts on the outline in the checklist?
- What are your thoughts on the three dimensions (background, methodology, results) of the checklist?
- Are the questions included in the checklist comprehensive?
- Is there a question that is less comprehensive?
- Do you understand the purpose and meaning behind each question?
- Is there a question for which the purpose or meaning could be made clearer or more explicit?
- Could you go over the questions with me and explain which question is relevant (or less relevant) for COI studies?
- Could you go over the questions with me and give me your feedback on the applicability of each question for COI studies?
- In your opinion, is there any question that is less relevant or applicable? Would you recommend removing this specific question from the checklist?
- In your opinion, is there anything missing in this checklist?
- Is there a question you would add to the checklist?
- In your opinion, is the checklist complete?
- How would you answer (or rate or score) the questions in the checklist? Any preference and why?
- What would you do with the answers?
- In your opinion, would you add up the answers? Please explain why (or why not)?
- What do you think about giving answers a score or weight? Why is this important (or not important)?
- What would you use this checklist for? (i.e., to conduct of a COI study, to review COI studies)
- Who would benefit from the implementation of this checklist?
- Would you recommend this checklist to other researchers, analysts, economists, or other?

*Closing*

- Is there anything else you would like to add?
- Is there anything else you would like to ask me?
